# D-Chiro-Inositol Treatment Affects Oocyte and Embryo Quality and Improves Glucose Intolerance in Both Aged Mice and Mouse Models of Polycystic Ovarian Syndrome

**DOI:** 10.3390/ijms21176049

**Published:** 2020-08-22

**Authors:** Eva Pericuesta, Ricardo Laguna-Barraza, Priscila Ramos-Ibeas, Julia L. Gutierrez-Arroyo, Juan A. Navarro, Katia Vera, Carlos Sanjuan, Elena Baixeras, Fernando Rodríguez de Fonseca, Alfonso Gutierrez-Adan

**Affiliations:** 1 Departamento de Reproducción Animal, Instituto Nacional de Investigación y Tecnología Agraria y Alimentaria (INIA), 28040 Madrid, Spain; pcamacho@inia.es (E.P.); rilaba@hotmail.com (R.L.-B.); ramos.priscila@inia.es (P.R.-I.); juliagut@ucm.es (J.L.G.-A.); 2 Laboratorio de Neuropsicofarmacología, Unidad de Gestión Clínica de Salud Mental, Instituto IBIMA, Hospital Regional Universitario de Málaga, 29010 Málaga, Spain; juan_naga@hotmail.es (J.A.N.); katiabm9@gmail.com (K.V.); fernando.rodriguez@ibima.eu (F.R.d.F.); 3 Euronutra S.L., Calle Johannes Kepler 3, 29590 Málaga, Spain; euronutra@euronutra.eu; 4 Departamento de Bioquímica y Biología Molecular, Facultad de Medicina, Universidad de Málaga, 29010 Málaga, Spain; ebaixeras@uma.es

**Keywords:** polycystic ovary syndrome, D-chiro-inositol, oocyte, embryo, fertility, oxidative stress, glucose intolerance

## Abstract

Polycystic ovarian syndrome (PCOS) is the main cause of female infertility. It is a multifactorial disorder with varying clinical manifestations including metabolic/endocrine abnormalities, hyperandrogenism, and ovarian cysts, among other conditions. D-*Chiro*-inositol (DCI) is the main treatment available for PCOS in humans. To address some of the mechanisms of this complex disorder and its treatment, this study examines the effect of DCI on reproduction during the development of different PCOS-associated phenotypes in aged females and two mouse models of PCOS. Aged females (8 months old) were treated or not (control) with DCI for 2 months. PCOS models were generated by treatment with dihydrotestosterone (DHT) on Days 16, 17, and 18 of gestation, or by testosterone propionate (TP) treatment on the first day of life. At two months of age, PCOS mice were treated with DCI for 2 months and their reproductive parameters analyzed. No effects of DCI treatment were produced on body weight or ovary/body weight ratio. However, treatment reduced the number of follicles with an atretic cyst-like appearance and improved embryo development in the PCOS models, and also increased implantation rates in both aged and PCOS mice. DCI modified the expression of genes related to oocyte quality, oxidative stress, and luteal sufficiency in cumulus-oocyte complexes (COCs) obtained from the aged and PCOS models. Further, the phosphorylation of AKT, a main metabolic sensor activated by insulin in the liver, was enhanced only in the DHT group, which was the only PCOS model showing glucose intolerance and AKT dephosphorylation. The effect of DCI in the TP model seemed mediated by its influence on oxidative stress and follicle insufficiency. Our results indicate that DCI works in preclinical models of PCOS and offer insight into its mechanism of action when used to treat this infertility-associated syndrome.

## 1. Introduction

PCOS is a multisystem reproductive-metabolic disorder that affects 5–25% of women of reproductive age causing ovulation and menstruation problems, hyperandrogenism, polycystic appearance of ovaries and, in most cases, infertility [1,2,3]. PCOS-associated metabolic dysfunction includes glucose and insulin resistance, dyslipidemia, type 2 diabetes mellitus, an increased risk of cardiovascular disease, and increasing prevalence of obesity [4]. PCOS is a complex disorder with a heterogeneous presentation and, so far, no animal model has been able to reflect all aspects of this human syndrome [5,6].

While the etiology of PCOS remains unknown, it can be caused by the overproduction of androgens in early life, and an excess of androgens has become the most widely used strategy to induce polycystic ovaries in animals [5]. Prenatal androgenization of female mice with dihydrotestosterone (DHT) causes irregular estrous cycles in adulthood [7,8] and affects metabolism by impairing glucose tolerance and pancreatic islet function but without causing obesity or peripheral insulin resistance [8]. As these DHT-induced PCOS mice show a modified reproductive biology but are not insulin resistant or hyperinsulinemic [8], they are a good model to assess the reproductive effects of D-chiro-inositol (DCI) that are independent of its insulin-lowering effect. This model reflects several features of women with a milder PCOS phenotype including glucose tolerance and an increased number of small antral follicles, but lacks cyst-like follicles and shows only mild adiposity with no change in adipokine levels [5]. Another model of PCOS can be produced by treating female mice with testosterone propionate (TP) during the first days of life, which leads to anovulation and poly-follicular ovaries [9], cyst-like follicles [10], and changes in follicular function later in life causing premature luteinization of follicles [11].

Ovarian aging with its complex gradual etiology also leads to a decrease in fertility. Several years before menopause, ovarian health declines asymptomatically, increasing ovarian failure and infertility in women [12]. Further, a perimenopausal age in women is associated with an increased prevalence of insulin resistance and obesity. Because of this complexity, the clinical diagnosis and treatment of infertility remain a challenge. Ovaries in middle-aged mice could be a good model to study ovarian aging. Considering that 42 years of human age is equivalent to 8 months of age in mice (http://www.age-converter.com/mouse-age-calculator.html), we selected this age for our aged mouse model.

In the past few decades, several therapies for human PCOS based on the administration of myoinositol (MI) and/or DCI [13] have been proposed. Inositol is a sugar represented by nine stereoisomers, of which MI (more than 99%) and DCI are the most widely distributed in the human body. MI and DCI are biologically active molecules known for their hypoglycemic actions, although they act through different mechanisms. MI regulates glucose transporters and glucose utilization, while DCI plays regulatory roles in glycogen synthesis (GS) and steroidogenesis, and is a component of a putative mediator of insulin action [14,15]. DCI has been also described to have anti-oxidation, anti-aging, and anti-inflammatory functions [16]. In the ovary, MI regulates glucose uptake and follicle-stimulating hormone (FSH) signaling, while DCI modulates insulin-induced androgen synthesis [17]. It has been also reported that DCI reduces blood pressure, plasma triglycerides and glucose concentrations, and improves ovarian function in women with PCOS [15,18]. DCI also reduces the production of reactive oxygen species (ROS) in the ovary, which are known to play a detrimental role in PCOS [19]. Moreover, DCI deficiency can lead to insulin resistance and as patients with PCOS show low plasma DCI levels, this highlights the correlation detected between plasma changes in DCI and insulin resistance [20]. In patients with type 2 diabetes mellitus (T2DM), the activity of epimerase, an enzyme that converts MI to DCI, is greatly reduced, effectively causing a decline in DCI synthesis [21]. Accordingly, DCI has been tested as a treatment for PCOS patients with hyperinsulinemia. In the first clinical trial of DCI (dose of 1200 mg) administered to women with PCOS over an 8-week period, ovulation was recorded in 86% compared to 27% for treatment with placebo [22]. Moreover, it seems that a high DCI dose can improve oocyte quality in women with PCOS undergoing assisted reproduction through intracytoplasmic sperm injection (ICSI) [23], and DCI has been also found to improve metabolic indices and ovarian function in PCOS patients [24]. The need to combine DCI with MI remains a matter of debate, especially when in vitro reproduction is considered to treat PCOS-infertility [25,26,27,28]. DCI has been also shown to be effective when used to treat some clinical manifestations of PCOS such as menstrual irregularity or anovulatory cycles [29,30]. To the best of our knowledge, however, the effects of DCI on the quality and gene expression of oocytes and preimplantation embryos have not been explored in mouse models of aging or PCOS. 

In this study, we first examined whether DCI could help resolve reproductive conditions associated with age and PCOS in aged female mice and models of DHT- and TP-induced PCOS also in mice. Next, we assessed the effects of DCI on the gene expression of cumulus-oocyte complexes (COCs) and blastocysts obtained from aged female, DHT-PCOS and TP-PCOS mice. Finally, as PCOS also produces metabolic dysfunctions, we explored whether the activation of AKT, a metabolic sensor modulated by pancreatic hormones and inflammation, could contribute to the beneficial effects of DCI in our mouse models.

## 2. Results

### 2.1. Effects of DCI on the Reproductive Biology and Glucose Tolerance of Aged Female Mice and Mouse Models of DHT-or TP-Induced PCOS 

Before starting the experiments, we checked for the presence of DCI in the diets used to feed the mice (Teklad Global Rodent Diets #2014 and #2018; Teklad Diets, Madison WI) by gas chromatography coupled to mass spectrometry (GC-MS) and gas chromatography coupled to a flame ionization detection (GC-FID). These analyses were able to confirm the absence of DCI in the mouse diets (Supplemental Table S1).

**Figure 1:**
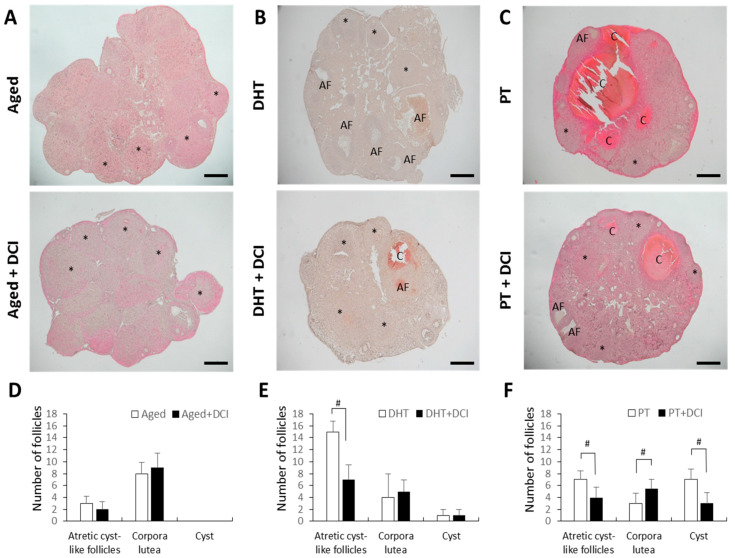
Morphological consequences of DCI treatment in aged and PCOS mouse ovaries. Mice were euthanized after superovulation on the day after HCG injection. (**A**) Histological analysis of ovarian sections from 8 month-old females (aged) after 2 months with (*n* = 5) or without DCI treatment (*n* = 6). Ovaries from mice with PCOS induced by DHT (**B**) or TP (**C**) both receiving (*n* = 7) or not (*n* = 6) DCI treatment. * Several (but not all) representative corpora lutea are shown in the image. (A,F): atretic cyst-like follicles; (**C**): cyst. Scale bar = 200 μm. (**D**–**F**) The mean number of atretic cyst-like follicles, corpora lutea and cysts recorded in the aged (**D**), DHT (**E**) and TP (**F**) groups. ^#^ *p* < 0.05.

Bodyweight did not differ between aged females (8 months) treated or not with DCI, and between females with PCOS treated or not with DCI. Ovaries from aged females treated with DCI or not had numerous corpora lutea consistent with recent ovulation, and showed no clear differences in morphology (Figure 1A,D). In PCOS ovaries, we observed different stages of developing follicles and increased numbers of follicles with a cystic-like appearance (Figure 1B,C,E,F). Ovaries from mice with DHT-induced PCOS had few corpora lutea, increased numbers of small antral follicles and several follicles with an atretic cyst-like appearance, but no clear cystic follicles (Figure 1B). A small number of cyst-like follicles were observed in ovaries from DHT-PCOS mice treated with DCI (Figure 1B lower and Figure 1E). Ovaries from mice with TP-induced PCOS had few corpora lutea, and showed several follicles with an atretic cyst-like appearance. However, when these mice were treated with DCI, their ovaries featured more corpora lutea and fewer cyst-like follicles (Figure 1C,F).

**Figure 2:**
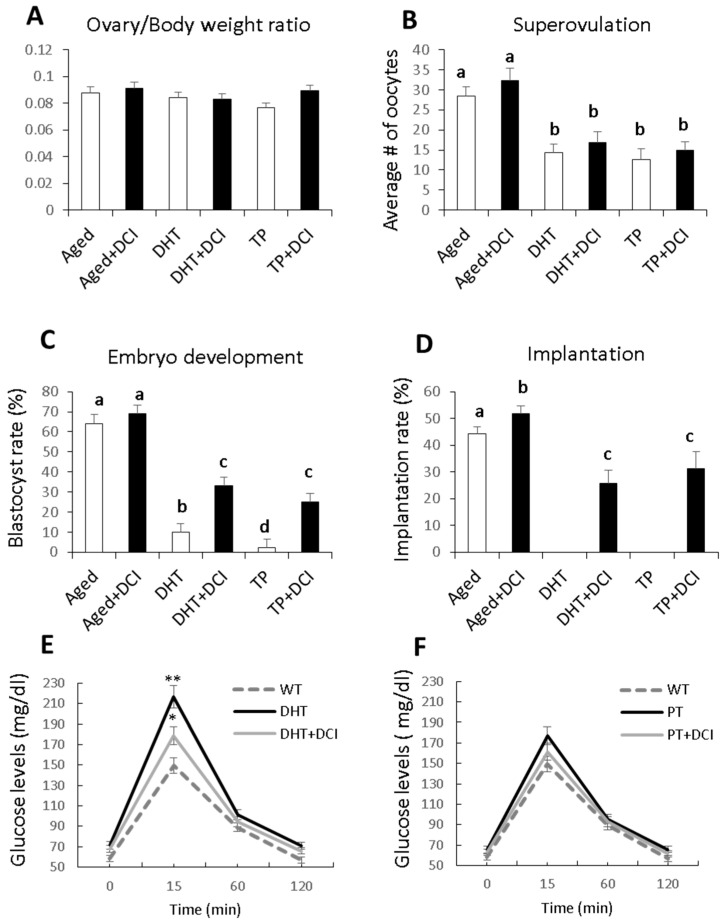
Influence of DCI treatment on reproductive parameters and glucose tolerance in aged and PCOS mouse models. (**A**) Ovary/body weight ratio (determined at euthanasia after superovulation). (**B**) Average number of oocytes retrieved from each mouse after superovulation and mating with males. (**C**) Percentage of embryos developing into blastocysts, and (**D**) percentage of blastocysts implanting in females after embryo transfer. (**E**,**F**) Glucose tolerance test (1.5 g glucose/kg body weight, i.p.) in WT control and PCOS mice of the same age after an overnight fast (*n* = 10 per group). Blood glucose measurements were taken every 15 min for 120 min with a blood glucose monitor (Accu-Chek). Glucose intolerance was observed in the DHT but not the TP model. DCI treatment reduced the effect of DHT; results provided as the mean ± SD. ^a,b,c,d^ different letters indicate significant differences (*p* < 0.05). *, *p* < 0.05; **, *p* < 0.01 compared with control mice.

Ovary/body weight ratios (determined at euthanasia after superovulation) were similar in females treated or not with DCI (*n* = 30 per group) (Figure 2A). Both models of PCOS showed reduced ovulation rates, which were significantly lower than in the aged female group. DCI treatment did not significantly improve the superovulation rate in any of the experimental groups (Figure 2B). Likewise, blastocyst rates were significantly reduced in the PCOS groups, the TP group being the most affected, and in both cases, DCI treatment improved embryonic development giving rise to more blastocysts (Figure 2C). While embryo implantation was fully impaired in both PCOS models, DCI treatment improved the implantation rate in the aged mouse group and allowed embryo implantation in the PCOS groups (Figure 2D). For the superovulation, embryo development, and implantation experiments, we did not use control groups without inducing PCOS because it is known that the CD1 strain has a good ovulation capacity, the in vitro development percentage is close to 90% and the implantation percentage of these embryos produced in vitro is higher than 50% [1], while here, in the experimental groups where we have induced PCOS, the embryonic development was very low and there were no implantations.

Random-feeding blood glucose levels did not differ among groups (data not shown), but fasting glucose was increased in the DHT-PCOS but not TP-PCOS females (Figure 2E,F) suggesting a prediabetic state of mice with DHT-induced PCOS. In addition, glucose tolerance was significantly lowered by DCI treatment in these DHT-PCOS mice (Figure 2E).

### 2.2. Effects of DCI Treatment on the Gene Expression Of Cumulus-Oocyte Complexes (COCs) and Blastocysts in Aged Female Mice and Models of DHT-and TP-Induced PCOS

**Figure 3:**
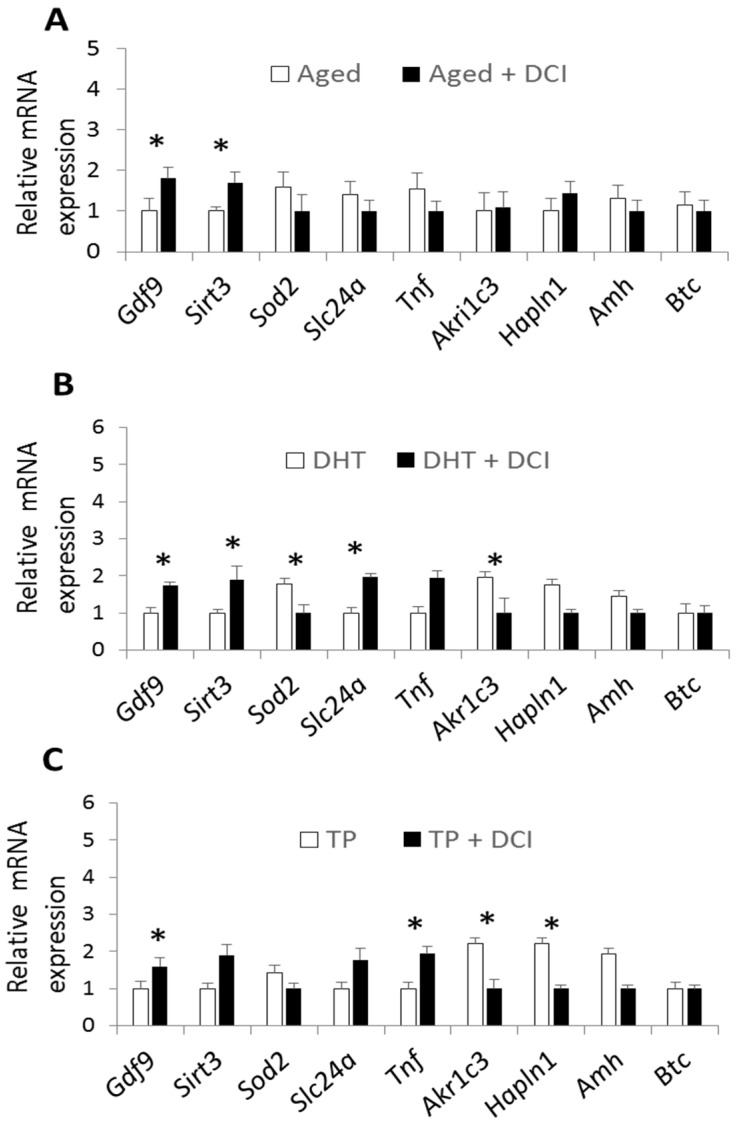
Effects of DCI treatment on relative mRNA transcription of selected genes in mouse oocytes with cumulus cells after superovulation. Relative expression in the aged group (**A**), DHT-PCOS group (**B**), and TP-PCOS group (**C**) treated or not with DCI. The genes analyzed are related to oocyte quality (*Gdf9*), oxidative stress (*Sirt3*, *Sod2*), glucose transporter (*Slc24a*), follicular angiogenic defects, COC expansion (*Tnf*, *Akr1c3*, *Hapln1*), anti-Müllerian hormone (*Amh*), and EGF-like ligand (*Btc*). Data are expressed relative to the housekeeping genes *Actb* and *H2afz*. Values are reported as the mean  ±  s.e.m. Significant differences in relative mRNA abundance between the control and experimental groups (*p*  <  0.05) are indicated with (*).

The expression of several genes was examined in COCs obtained from the three experimental groups: genes related to oocyte quality (*Gdf9*), oxidative stress (*Sirt3*, *Sod2*), glucose transporter (*Slc24a*), follicular angiogenic defects and COC expansion (*Tnf*, *Akr1c3*, *Hapln1*), anti-Müllerian hormone (*Amh*), and EGF-like ligand (*Btc*). In the aged mouse group, DCI treatment led to a significantly increased expression of *Gdf9* and *Sirt3* (Figure 3A). In the DHT-PCOS model, DCI increased the expression of *Gdf9*, *Sirt3* and *Slc24a*, and reduced expression levels of *Sod2* and *Akr1c3* (Figure 3B). Finally, in the TP-PCOS model, DCI increased *Gdf9* and *Tnf* expression, and reduced *Akr1c3* and *Hapln1* expression (Figure 3C).

**Figure 4:**
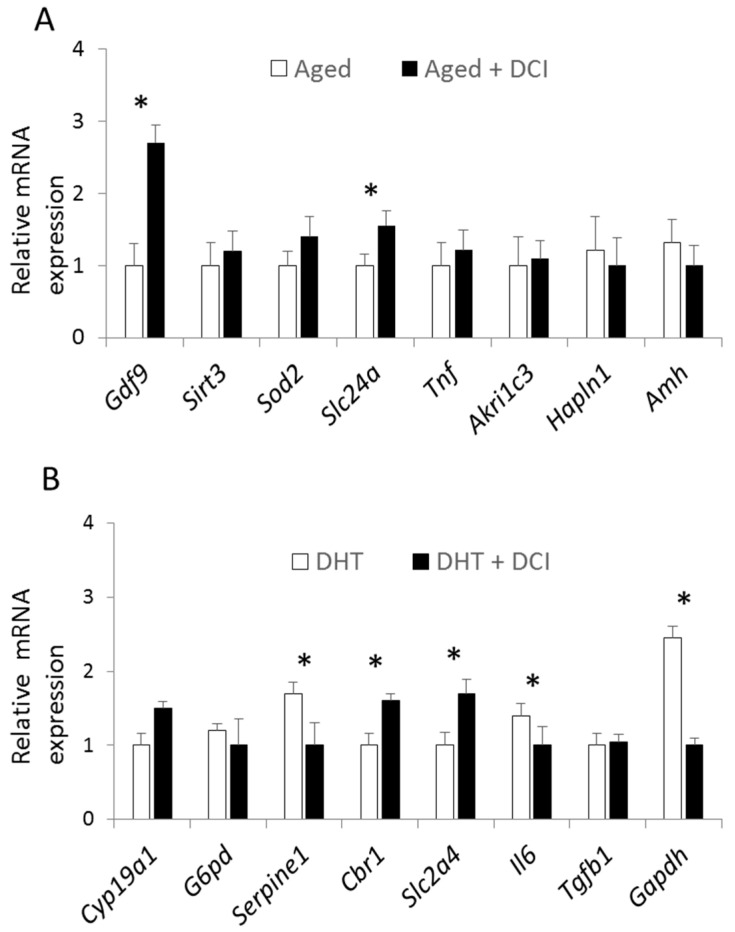
Effect of DCI treatment on relative mRNA transcription of selected genes in mouse blastocysts after in vitro culture. Relative expression in the aged group (**A**) and DHT-PCOS group (**B**) treated or not with DCI. The genes analyzed were those related to estradiol and estrone biosynthesis (*Cyp19a1*), glucose transport and metabolism (*G6pd*, *Slc2a4*, *Gapdh*), serine protease inhibitor linked to PCOS (*Serpine1*), oxidative stress (*Sirt3*, *Sod2*), follicular angiogenic defects, COC expansion (*Tnf*, *Akr1c3*, *Hapln1*), anti-Müllerian hormone (*Amh*), pro-inflammatory cytokine (*Il6*), and transforming growth factor (*Tgfb1*). Data are expressed relative to the housekeeping genes *Actb* and *H2afz*. Values are reported as the mean  ±  s.e.m. Significant differences in relative mRNA abundance between the control and experimental groups (*p*  <  0.05) are indicated with (*).

Gene expression at the blastocyst stage was only examined in aged mice and in the DHT-induced PCOS model as insufficient numbers of blastocysts were produced in the TP model. DCI supplementation in the aged females increased the expression of *Cyp19a1* and *Cbr1* (Figure 4A). Blastocysts from the DHT-PCOS model treated with DCI showed the increased expression of *Cbr1* and *Slc2a4*, and reduced expression of *Serpine1*, *Il6*, and *Gapdh* (Figure 4B).

### 2.3. Effects of DCI on AKT Phosphorylation in the Liver of Mice with PCOS

**Figure 5:**
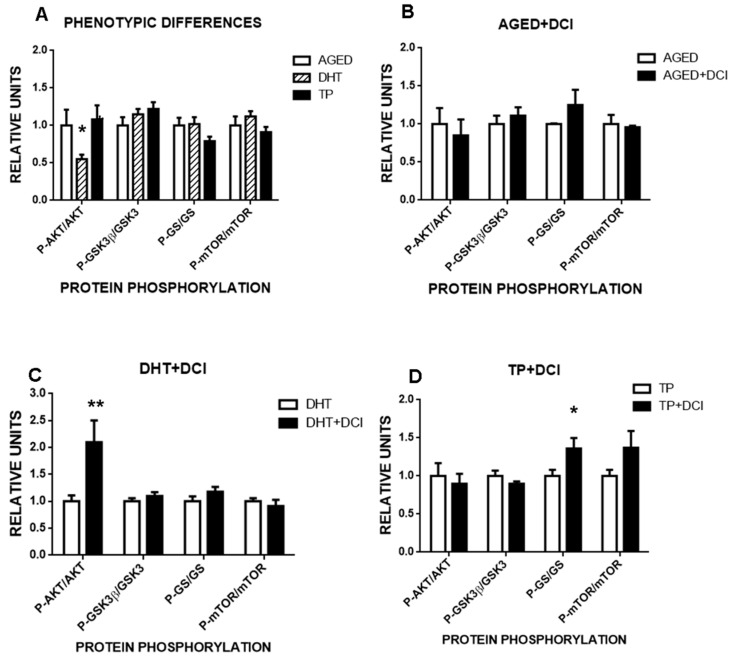
Influence of DCI treatment on the phosphorylation state of the liver metabolic sensors AKT and mTOR, and on that of the glycogen synthesis-related enzymes GSK3β and GS in the aged and PCOS mouse models. (**A**) Normalized phosphorylation state measured as the ratio between phosphorylated/non-phosphorylated forms of the proteins AKT (protein kinase B), GSK3β (glycogen synthase kinase 3-beta), GS (glycogen synthase) and mTOR (mammalian target of rapamycin) in the liver of adult aged animals, or adult animals born from mothers treated with dihydrotestosterone (DHT) or testosterone propionate (TP). Effects of 2 months of supplementation with DCI (20 mg/kg) on the phosphorylation state of the same proteins in (**B**) aged mice, (**C**) DHT-treated mice, and (**D**) TP-treated mice. Values are reported as the mean  ±  s.e.m. Significant differences between the control and experimental groups (*p  <  0.05, **p  <  0.01).

To test whether glucose intolerance was the outcome of a metabolic imbalance, we selected two metabolic sensors associated with insulin resistance and obesity (the kinases AKT and mTOR), and two proteins associated with glycogen synthesis, glycogen synthase kinase-3 (GSK-3) and glycogen synthase (GS) (Supplementary Figure S1). We found that animals exposed to DHT, but not to TP, had lower phosphorylated AKT levels, suggesting lower activation of this sensor (interaction experimental group (aged, DHT or TP) x treatment (vehicle or DCI), F(6,86) = 2.45, *p* < 0.03, Figure 5A). This lowering of AKT phosphorylation in the liver suggests reduced insulin signaling, which could promote glucose intolerance as seen in the glucose tolerance test. As remaining proteins were in the normal phosphorylated state, we propose that a compensatory mechanism could explain the normal basal glycemia observed in the DHT-PCOS animals. DCI treatment resolved this AKT phosphorylation deficit, doubling phosphorylated AKT levels (interaction experimental group x treatment, F(3,59) = 3.32, *p* < 0.03, Figure 5C). With respect to glycogen synthesis, DCI was in general capable of increasing GS phosphorylation, although this effect was only significant in the TP-PCOS group (simple effect of treatment F(1,18) = 7.5, *p* < 0.02, Figure 5B–D).

## 3. Discussion

Our study reveals the effects of two months of treatment with DCI on ovary histology, superovulation, embryo production, implantation rate, and oocyte and blastocyst gene expression levels in aging female mice, and two models of PCOS created also in mice. While the benefits of DCI treatment in women with PCOS have been confirmed in clinical practice, the mechanisms whereby DCI improves reproductive parameters remain unclear, and conclusive data are lacking regarding the need for co-supplementation with MI. Although no animal model can perfectly replicate this human disorder, the similarity of some phenotypes of PCOS animal models with women with PCOS suggests several common pathophysiological features. We found here that DHT-treated fetal mice and mice treated with TP on their day of birth had reproductive abnormalities that mimicked symptoms of human PCOS. However, only the fetal mice with DHT-induced PCOS featured glucose resistance, suggesting that this is a good model for the study of metabolic abnormalities in PCOS, whereas the TP model is good for analyzing PCOS features not related to glucose metabolism. This was also reflected by the selective liver dephosphorylation of AKT, a metabolic sensor activated by insulin, which was partially deactivated in the DHT-treated mice, but not in the TP-treated ones. Moreover, both models gave rise to different histological modifications in the ovary and a reduced quality of blastocysts. While most pronounced effects were observed in the TP-model, blastocysts had the capacity for implantation in both models. DCI treatment was able to rescue most of the reproductive/metabolic impacts observed. It should be noted that we used DCI-free diets so we could monitor the effects of exogenous DCI supplementation. DCI treatment modified gene expression in COCs and blastocysts from both models in different ways, and while the total production of oocytes or embryos was unaffected, DCI treatment allowed for the implantation and development of the embryos. Moreover, DCI probably improved glucose intolerance in the DHT model animals by increasing the activity of AKT in the liver, as revealed by our phosphorylation studies. Since this is the classic insulin-mimetic action of DCI, it is reasonable to speculate that DCI might mimic insulin in other tissues, leading to a general metabolic improvement that favors reproduction, as reflected by the improved implantation rate, driven possibly by PI3K-AKT-induced genes that act as an embryo surviving factor [31]. Another well-known effect of DCI is its capacity to enhance glycogen synthase (GS) activity and glycogen deposits, which could be observed here as a mild general treatment effect independent of metabolic status.

Fertility decreases dramatically in women older than 40 years. For this reason, we used females that were 8 months old in our aged mouse model, which is equivalent to 42 years of human age. In humans, the prevalence of metabolic dysfunctions is also high [32], and a decrease in egg quality is associated with a gradual increase in circulating levels of follicle-stimulating hormones and with a decrease in circulating anti-Müllerian hormone [33]. Inositols may have a potential role to play in maintaining metabolic health and increasing fertility in middle aged women [32]. Here, we found that DCI treatment had no effect on the production of oocytes or blastocysts, but did have a positive effect on implantation, suggesting this treatment could increase oocyte and blastocyst quality. Our gene expression analysis indicated that DCI increased the expression of Gdf9, a gene known to play a role in early ovarian folliculogenesis, which confers developmental competence to the blastocyst stage [34]. DCI was also found to increase expression levels of *Sirt3*, a gene that helps control ROS homeostasis in oocytes. Increased ROS levels and decreased *Sirt3* expression have been also observed in oocytes harvested from diabetic mice [35]. Our results suggest that DCI plays a protective role against oxidative stress in oocytes from aged females, and this also has a positive effect on *Gdf9* expression. At the blastocyst stage, DCI increased the expression of *Cyp19a1*, a gene encoding aromatase that is downregulated in parallel with advancing age [36]. A role for the aromatase isoform present in pig and horse blastocysts has been proposed in the synthesis of estrogens that induce peri-implantation embryo-maternal signaling [37]. We also observed that DCI increases the expression of *Cbr1*, a prostaglandin 9-ketoreductase which converts prostaglandin E2 into prostaglandin E2a, whose expression in peri-implantation pig embryos has been reported to promote pregnancy [38]. Thus, the upregulation of *Cyp19a1* and *Cbr1* induced by DCI supplementation could be related to the increased implantation observed. Collectively, these findings support the use of DCI as a nutritional supplement for healthy reproductive aging.

To address the effects of DCI in PCOS, we generated two mouse models of this syndrome. In our first model of prenatally induced PCOS based on the use of DHT, we aimed for a milder phenotype of PCOS [7]. In this model, we confirmed that DCI treatment did not modify body mass but altered ovarian morphology and embryo production, development and implantation, and also induced glucose intolerance. DCI supplementation increased embryo development and implantation, and modified gene expression in COCs and blastocysts. This treatment increased expression levels of mRNA for *Fdf9* and *Sirt3*, and reduced those for *Sod2* in oocytes, suggesting a beneficial effect on oocyte quality and oxidative stress. Alterations in *Sod2* expression have been reported in oocytes of different mammals under different conditions [39,40]. At the oocyte level, DCI is the substrate of phosphatidylinositol-3-kinase (PI3K), an enzyme involved in oocyte activation and in the survival and activity of granulosa cells on which oocyte quality depends, and is crucial for oocyte and preimplantation development [41]. Further, we noted that DCI increased the expression of *Slc24a*, an insulin-dependent glucose transporter [42] located in cumulus cells and associated with COC energy metabolism [43]. To further analyze the signaling pathways of DCI, we selected the liver to assess the phosphorylation state of AKT, a metabolic sensor controlling glycolysis, glycogen synthesis, and glucose export. According to our data, DCI was able to rescue the deficiency of AKT activity (measured as activating phosphorylation) induced by DHT. As DCI has been also described in striate muscle [44,45], we speculate that if present in other tissues, DCI could restore the PI3k-AKT pathway and help counteract the effects of PCOS syndrome. Interestingly, other metabolic sensors such as mTOR were not noted to play a role in this response to DCI. Since mTOR is capable of modulating the activity of the insulin receptor [46], the lack of changes detected in the phosphorylation state of mTOR suggests that the effects of DCI appear downstream from the insulin receptor. This observation is in line with reports suggesting that the insulin-mimicking actions of DCI are not dependent on this receptor.

When there is insulin resistance, it seems that the conversion rate of MI into DCI is compromised, reducing cellular levels of DCI [47]. Accordingly, the exogenous administration of DCI may enhance the activity of the insulin-receptor, reducing glucose levels [47]. DCI has been shown to have insulin-like bioactivity and to reduce meal-induced hyperglycemia [48]. Moreover, DCI has been described to decrease the expression of *Akr1c3*, a gene found upregulated in the granulosa cells of women with PCOS [49]. ADR1C3 is a steroidogenic enzyme that converts androstenedione (A4) into biologically active testosterone in non-testicular tissues [50]. Moreover, AKR1C3 also acts as a prostaglandin F synthase to facilitate luteolysis [50], and luteal insufficiency and premature luteolysis are frequent in PCOS [49]. Interestingly, a relationship has been detected between *Sirt3* and *Sod2* expression and AKT phosphorylation, protecting oocytes against oxidative stress in diabetic mice [35]. Moreover, AKT substrates trigger the translocation of SLC2A4 to the plasma membrane, resulting in glucose uptake [42], and its phosphorylation can be induced by *Akr1c3* [51]. Thus, DCI effects on AKT phosphorylation can give rise to beneficial impacts on the expression of genes related to glucose tolerance, oxidative stress, and hormone metabolism.

In blastocysts of our DHT model, besides the effects of DCI treatment on *Cbr1* mRNA expression also observed in the aged mouse model, DCI reduced the expression of *Serpine1*, *Il6*, and *Gapdh*, and increased the expression of *Slc2a4*. The up-regulation of *Serpine1* has been reported in bovine blastocysts under hyperglycemic conditions, suggesting that the increased activity of the hexosamine pathway could be a marker of developmentally compromised embryos [52]. The up-regulation of *Serpine1* in granulosa cells has also been described in women with PCOS [53], but its expression in blastocysts obtained from PCOS patients remains unknown. Our results indicate, however, that DCI treatment reduced *Serpine1* expression in blastocysts derived from our DHT-PCOS model. DCI also reduced the expression of *Gapdh*, a glycolytic enzyme that also participates in numerous cellular functions such as exocytosis, cytoskeletal organization, DNA replication and repair, endocytosis, iron metabolism, carcinogenesis, and cell death [54]. As insulin increases mRNA levels of *Gapdh*, we suggest that the effect of DCI in blastocysts from the DHT-PCOS model could be related to a beneficial effect on glucose metabolism. DCI also reduced the expression of *Il6* mRNA in the DHT model. *Il6* seems to be hormonally modulated in mouse blastocysts during implantation [55]. Furthermore, it has been reported that granulosa cells from patients with PCOS express elevated levels of transcripts coding for cytokines like *Il6*, that DHT increases cytokine production, and that *Il6* expression is significantly associated with a pregnancy event in women with and without PCOS [56].

Because PCOS manifests heterogeneously in women, we produced an alternative PCOS model induced by testosterone propionate injected on the first day of life [57]. Testosterone has often been used in rat models of PCOS, but not so frequently in mice. In early work, treatment of female mice during the first 3 days of life was found to give rise to anovulation and poly-follicular cystic ovaries [9]. In rats, this method has been used to assess the effects of androgens on follicular physiology, likely due to epigenetic modifications [58]. For a less drastic treatment, we used only one TP injection. Our treatment reduced the number of oocytes obtained by superovulation and had a greater effect on the development of the embryos than the DHT model, while embryo implantation was completely impaired. Histological analysis of the ovary revealed differences with the DHT model, like the development of follicular cysts and fewer luteal bodies. In addition, this model did not lead to glucose intolerance. We were unable to obtain sufficient numbers of blastocysts for mRNA expression analysis, but COCs treated with DCI in this TP model showed higher expression of Gdf9 and Tnf, and lower expression of Akr1c3 and Hapln1. In agreement with our results, transcripts of AKR1C3 and HAPLN1 have been found upregulated, while Tnf was downregulated in the granulosa cells of women with PCOS [49]. The pro-inflammatory cytokine Tnf suppresses FSH-induced Lhcgr promoter activation and could be a factor contributing to hyperandrogenemia. Moreover, optimal TNF levels have been reported to confer a protective function in the maintenance of bovine granulosa cells and oocytes and to facilitate ovulation [49,59], while lowered TNF in the granulosa cells of women with PCOS may hamper COC expansion, compromising ovulation [49]. AKR1C3 is a steroidogenic enzyme that converts androstenedione into testosterone in non-testicular tissues, and is expressed by the granulosa cells of periovulatory follicles [60]. Its higher expression in the granulosa cells of women with PCOS may contribute to high androgen production in their ovaries [49]. Moreover, AKR1C3 acts as a prostaglandin F synthase, which is important for luteal function, and premature luteolysis frequently occurs in PCOS [49]. Our results suggest that the beneficial effect of DCI in our TP-PCOS model could target COC matrix expansion defects, luteal insufficiency, and/or androgen-excess.

Our mRNA expression results for the oocytes and embryos of our aged and PCOS mouse models treated with DCI suggest that, besides its glycemic effects, this treatment may affect other metabolic systems, attenuating the clinical manifestations of PCOS.

## 4. Materials and Methods

### 4.1. Animals

All reagents were purchased from Sigma (Madrid, Spain) unless indicated otherwise. Animal experiments were conducted following European legislation. All study protocols were approved by the Ethical Committee on Animal Experimentation of the INIA (Madrid, Spain) (21 September 2015) and was registered on the Direccion General de Agricultura y Ganaderia de la Comunidad de Madrid (Spain) (PROEX 261/15, 4 November 2015). Written informed consent was obtained from all participants. CD-1 mice were used for all the experimental models (CD-1®, Envigo, Horst, The Netherlands). Mice were provided the standard diet Teklad Global Rodent Diets #2014 during the 2 first months (Harlan Iberica, Sant Feliu de Codines, Catalonia, Spain) of life and then #2018; (Teklad Diets, Madison, WI, USA) ad libitum and kept in a temperature- and light-controlled environment (22–24 °C, light:dark 14:10 h). For the aged female experiments, CD-1 mice were housed (6–8 per cage) for 6 months, and then females were randomly divided into two groups, control (*n* = 60) and DCI (*n* = 60). DCI (Caromax, Euronutra, Malaga, Spain) was administered in drinking water at a dose of 150 mg/L per day as indicated by the supplying company (Euronutra) (based on measured water consumption of approximately 4 mL/day by a 30 g mouse; this equates to approximately 20 mg/kg per day).

Cumulus-oocyte complexes (COCs) were collected from the female aged and PCOS mouse models after superovulation with 7.5 IU of equine chorionic gonadotropin (Folligon 500, Intervet, Madrid, Spain) followed by 7.5 IU of hCG (Veterin Corion, Equinvest, Lisbon, Portugal) 48 h later. The embryos were cultured in 20 µL drops of equilibrated culture medium KSOMaa (Evolve; Zenith Biotech, Guilford, CT, USA), overlaid with mineral oil at 37 °C under an atmosphere of 5% CO_2_ in air with maximum humidity. Embryos were cultured for 5 d; and cleavage rates assessed on Day 1 (24 h after fertilization) and blastocysts on Day 4 (96–100 h after fertilization).

To determine implantation rates, 2-cell embryos were transferred to the left fallopian tube 0.5 days post coitum [61]. On the morning of Day 12, the mice were anesthetized by CO_2_ exposure followed by cervical dislocation, and uteri were examined for embryo implantation sites. The percentage of implantations on Day 12 was referred to the number of embryos transferred.

### 4.2. Generation of PCOS Mouse Models

To generate the DHT-PCOS model, we used 2 month-old adult CD-1 females. Females were paired with males and checked for copulatory plugs. The date of the plug was recorded as Day 1 of gestation. Pregnant mice were injected with 50 µL sesame oil containing 250 g of DHT on Days 16, 17, and 18 of gestation. For the TP-PCOS model, CD-1 females were injected on their day of birth with 100 µg of TP diluted in oil, and control mice were treated with oil only. All mice were allowed free access to their mothers until weaning (Day 24). Thereafter, they were allowed free access to water and food until the day of euthanasia.

### 4.3. Superovulation Study

Adult female mice of various genotypes (6 to 15 weeks old) were intraperitoneal injected (i.p.) with pregnant mare serum gonadotropin (PMSG, 5 IU; Calbiochem/MilliporeSigma, Burlington, MA, USA) followed by an i.p. of human chorionic gonadotropin (hCG) 48 h later (5 IU; Calbiochem). The animals were euthanized 14 h after the hCG injection. Bodyweight and ovarian weight were recorded. Ovulated oocytes were collected from the ampulla of the oviduct and counted.

### 4.4. Chromatographic Determination of DCI in the Diets Used as Treatment

DCI was determined in the mice diets used (Teklad Global Rodent Diets #2018 and #2018; Teklad Diets, Madison, WI, USA) by gas chromatography coupled to mass spectrometry (GC-MS) and gas chromatography coupled to a flame ionization detection (GC-FID).

GC-FID analysis was performed following the method of Cardelle-Cobas et al. [62] using an Agilent Technologies 7890A gas chromatography instrument (Agilent Technologies, Wilmington, DE, USA) equipped with a flame ionization detector (FID). Separations were carried out using a fused silica capillary column HP-5MS (5% phenylmethyl silicone, 25 m × 0.32 mm × 0.25 μm thickness; J & W Scientific, Folsom CA, USA). Nitrogen was used as the carrier gas at a flow rate of 1 mL min^−1^. Injector and detector temperatures were 280 and 315 °C, respectively. The oven temperature was programed from 180 to 315 °C at a heating rate of 3 °C min^−1^ and held for 60 min. Injections were made in the split mode (1:20). Data acquisition and integration were conducted using Agilent ChemStations Reb. 4B. 03.01 software (Wilmington, DE, USA). All analyses were run in duplicate. Response factors were calculated after the triplicate analysis of 5 standard solutions (myoinositol, galactose, glucose, fructose, sucrose, raffinose, and stachyose) over the expected concentration range in the samples (0.01–1 mg mL^−1^).

GC-MS analysis was performed in an Agilent Technologies 7890A gas chromatography device coupled to a 5975CMSD quadrupole mass detector (Agilent Technologies, Palo Alto, CA, USA) to confirm the identification of all carbohydrates [63]. Sugar separation was performed using helium as the carrier gas at 0.8 mL min^−1^. Separations were carried out using a fused silica capillary column HP-5MS (5% phenylmethyl silicone, 25 m × 0.32 mm × 0.25 μm thickness; J & W Scientific, Folsom CA, USA). The injector temperature was 280 °C. The oven temperature was programmed from 180 to 315 °C at a heating rate of 3 °C min^−1^ and held for 60 min. Injections were made in the split mode (1:20). The mass spectrometer was operated in electrospray ionization mode at 70 eV. Mass spectra were acquired using Agilent ChemStation MSD software (Wilmington, DE, USA). Trimethylsilyl oxime derivatives of carbohydrates were identified through comparison of their relative retention times and mass spectra with those of previously derivatized standard compounds.

### 4.5. Histological Analysis

Ovaries were collected from 2-month-old females and fixed in 4% paraformaldehyde in PBS at 4 °C overnight. These samples were dehydrated through an ethanol gradient, embedded in paraffin wax, and serially sectioned at a thickness of 6 µm. Sections at 60-µm intervals (every 10th section) were stained for systematic histological analysis with hematoxylin and eosin and scanned using an Aperio ScanScope XT Scanner (Aperio Technologies/Leica Microsystems, Buffalo Grove, IL, USA) for digital image analysis [64]. The number of follicles (atretic cyst-like follicles, antral follicles, and corpora lutea) were counted in every tenth section and multiplied by 10 to give the total number of follicles in each ovary (4 females per group). Only follicles containing an oocyte with a visible nucleus were counted to avoid double counting. The number of corpora lutea was scored in a blinded fashion on one section per ovary and one ovary per mouse.

### 4.6. Analysis of mRNA Levels by RT-qPCR

Messenger RNA was extracted from 3 pools of 10 oocytes or 10 embryos at the blastocyst stage using the Dynabeads mRNA Direct Extraction KIT (Dynal Biotech, Madrid, Spain) according to the manufacturer’s instructions. Immediately after extraction, the reverse transcription (RT) reaction was performed with the BioTaq enzyme (Bioline, London, UK) according to the manufacturer’s instructions. To prime the RT reaction and synthesize cDNA, poly(T) primer, random primers, and Moloney murine leukemia virus (MMLV) reverse transcriptase enzyme were used in a total volume of 40 µL. Tubes were heated to 70 °C for 5 min to denature the secondary RNA structure and the RT reaction was completed with the addition of 100 units of reverse transcriptase. The mixture was incubated at 42 °C for 60 min to allow the RT of RNA, which was followed by incubation at 70 °C for 10 min to denature the RT enzyme [65]. Three groups of cDNA were set up for each experimental group with two replicates for all genes of interest. PCR was performed by adding a 2 μL aliquot of each sample to the PCR mix containing specific primers to amplify the genes of interest. Primer sequences are provided in Supplementary Table S2. Expression levels were normalized against that of the endogenous control H2afz as described previously [66]. PCR conditions were optimized to achieve efficiencies close to 1. The comparative cycle threshold (CT) method was used to quantify expression levels. Fluorescence was acquired in each cycle to determine the threshold cycle or the cycle during the log-linear phase of the reaction at which fluorescence increased above the background level for each sample. Within this region of the amplification curve, a difference of one cycle is equivalent to doubling of the amplified PCR product. According to the comparative CT method, ΔCT was determined by subtracting the average CT value obtained for the control genes (*H2afz* and *Actb*) from the CT value for each gene of interest in each sample. To calculate ΔΔCT, the highest sample ΔCT value (i.e., the sample showing the lowest target expression) was used as an arbitrary constant to subtract from all other ΔCT sample values. Fold changes in relative gene expression levels of target genes were determined using the formula 2−ΔΔCT [67]. Gene expression levels were then normalized to those of *Actb* and *H2afz*.

### 4.7. Western Blots

Western blot analysis of the phosphorylation state of AKT, mTOR, CSK3B, and GS was done according to published methods [68]. Total protein from 15–25 mg of liver samples was extracted using an ice-cold cell lysis buffer for 30 min. Fifty micrograms of protein were resolved on 12% (Bis-Tris) Criterion XT Precast Gels (Bio-Rad Laboratories, Inc., cat. number: 3450124, Madrid, Spain) and electroblotted onto nitrocellulose membranes (BioRad). For specific protein detection, the membrane was incubated 1 h in TBS-T containing 2% BSA and the corresponding primary antibody. The phosphorylated form of proteins was determined using the corresponding rabbit anti-phospho-AKT, phospho-GSK3β, phospho-glycogen synthase, and phospho-mTOR (Cell Signaling Technology Inc., Beverly, MA, USA). Total protein was detected using rabbit anti-AKT, anti-GSK3β, anti-glycogen synthase, and anti-mTOR, respectively (Cell Signaling Technology Inc.). Adaptin γ was detected using mouse anti-adaptin γ (Becton, Dickinson, and Company (BD), Franklin Lakes, N.J., USA). Primary antibodies were detected using anti-rabbit or an anti-mouse HRP-conjugated antibody as appropriate (Promega, Madison, WI, USA, respectively). Specific proteins were revealed using ECL™ Prime Western Blotting System (GE Healthcare, Chicago, IL, USA) according to the manufacturer’s instructions. Images were visualized in a ChemiDoc MP Imaging System (Bio-Rad, Hercules, CA, USA). After measuring phosphorylation proteins, specific antibodies were removed from the membrane by incubation with stripping buffer (2% SDS, 62.5 mM Tris HCL pH 6.8, 0.8% ß-mercaptoethanol) for 30 min at 50 °C. Membranes were thoroughly washed in ultrapure water and then re-incubated with the corresponding antibody specific for total protein. Results were quantified using ImageJ software (http://imagej.nih.gov/ij). The specific signal level for total proteins was normalized to the signal the level of the corresponding adaptin γ band in each sample and same blot. The phosphorylation stage of a protein was expressed as the ratio of the signal obtained with the phosphospecific antibody relative to the appropriate total protein antibody. The amounts of the protein of interest in control samples were arbitrarily set at 1. Unedited gel blots can be found as supplementary material.

### 4.8. Glucose Blood Test

Mice were transferred to a metabolic clean cage and fasted overnight (16 h) with ad libitum access to water. In the morning, they were subjected to a blood glucose tolerance test. Whole-blood b-D-glucose levels were determined using a standard handheld glucometer (Glucocard G-sensor, Arkray Factory, Inc, Shiga, Japan) in blood samples (2 µL/measure) collected from the tip of the tail. Following baseline glucose measurements, mice were injected i.p. with glucose (20% solution, 1.5 mg/g) and blood glucose readings were then taken 15, 60, and 120 min post-injection. Food was reintroduced immediately after the last reading. The area under the curve (AUC) was calculated for suprabasal plasma glucose (AUCglucose) levels, for the entire 120-min study period applying the trapezoid rule.

### 4.9. Statistical Analysis

For comparisons of ovarian weight, oocyte counts after superovulation, number of atretic cyst-like follicles, corpora lutea, and cysts, an unpaired t-test with equal SD was used. Cleavage rates, blastocyst yields, relative mRNA abundances for candidate genes, and implantation rates were compared by one-way ANOVA followed by multiple pairwise comparisons using the Tukey post hoc test for most data. A minimum of three biological replicates were run in each experiment. All statistical tests were performed using the software package SigmaStat 3.5 (Jandel Scientific, San Rafael, CA, USA). Significance was set at *p* < 0.05.

## Supplementary Materials

Supplementary materials can be found at https://www.mdpi.com/1422-0067/21/17/6049/s1.

## Abbreviations

PCOS	Polycystic ovarian syndrome
DCI	D-Chiro-inositol
DHT	Dihydrotestosterone
TP	Testosterone propionate
COCs	Cumulus-oocyte complexes
Mi	Myoinositol
Gs	Glycogen synthesis
FSH	Follicle-stimulating hormone
ROS	Reactive oxygen species
T2DM	Type 2 diabetes mellitus
ICSI	Intracytoplasmic sperm injection
GC-MS	Gas chromatography coupled to mass spectrometry
GC-FID	Gas chromatography coupled to a flame ionization detection
hCG	Human chorionic gonadotropin
FID	Flame ionization detector
